# Ghost cell odontogenic carcinoma of the maxilla: a case report with a literature review

**DOI:** 10.11604/pamj.2015.21.260.5139

**Published:** 2015-08-07

**Authors:** Elneel Ahmed Mohamed Ali, Musadak Ali karrar, Abeer Abdalla El-Siddig, Nahla Gafer, Ali Abdel satir

**Affiliations:** 1 Oral & Maxillofacial Surgery, Faculty of Dentistry, University of Khartoum, Khartoum, Sudan; 2 OMF Surgery, National University, Khartoum, Sudan; 3 Faculty Of Medicine, Taibah University, KSA; 4 Radiation and Isotope Khartoum Center, Khartoum, Sudan; 5 Department of Pathology, Faculty of Medicine, University of Khartoum, Khartoum, Sudan

**Keywords:** Odontogenic carcinoma, ghost cell, mandible, Sudan

## Abstract

The aim of this report is to present a rare case of a Ghost cell odontogenic carcinoma (GCOC) of a 21-year-old man with review of the literature. The patient was treated surgically, and one of the surgical margins was involved, the patient received adjuvant radiotherapy for local control. Five months later, patient presented with infraorbital lesion which was proven histological to be GCOC. Radiological images, histological sections and clinical photographs are also presented. One year after the second surgery, the patient was tumor free. The only effective treatment modality was surgical removal.

## Introduction

Ghost cell odontogenic carcinoma (GCOC) is extremely rare with only 30 case reports in the English literature [1]. This disease has unpredictable biological behavior [2] which may arise de novo or from preexisting calcifying cystic odontogenic tumor (CCOT) [3]. GCOC was so called because of the presence of keratinized ghost cells in association with ameloblastomatous epithelial component and a CCOT with frank malignant counterpart. In general odontogenic tumors are rare neoplasms when compared to non-odontogenic tumors in the oral cavity. According to Adebayo et al [4], the odontogenic neoplasms represent (32%) of tumors and tumor-like lesions of the oral and perioral structures. Of these, malignant odontogenic carcinomas represent 1%.

## Patient and observation

A 21 year old male non tobacco user with a history of surgical removal of a small mass started after tooth extraction. The swelling reappeared again, and the patient was referred from a peripheral dental clinic to the department of Oral & Maxillofacial Surgery-Khartoum Teaching Dental Hospital. He was complaining of swelling at the left side of the maxilla of 2-years duration and difficulties in eating and mouth closure associated with mild pain and facial deformity. The swelling increased gradually in size with itching sensation and teeth mobility. The past medical history, family history, personal history and drug history were noncontributory. On extra oral examination there was a solitary, bony hard, painless, diffuse swelling of approximately 10x10 cm on the left side of the face obliterating the nasolabial fold with intact overlying skin. Careful clinical examination did not reveal any occult lesions or cervical lymphadenopathy

**Figure 1:**
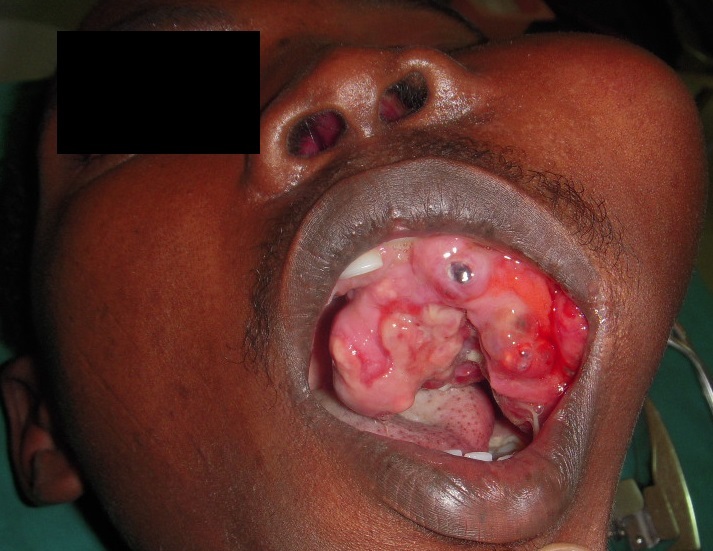
Diffused swelling on the left side of face obliterating the nasolabial fold

**Figure 2:**
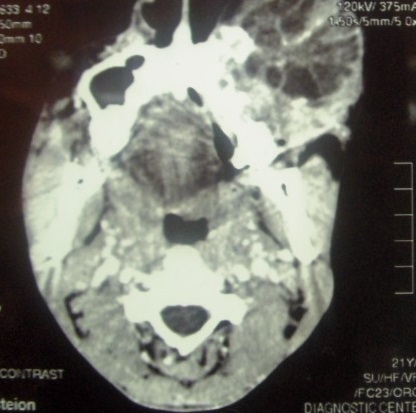
Axial section CT scan showed left maxillary expansion with mixed radiolucency

**Figure 3:**
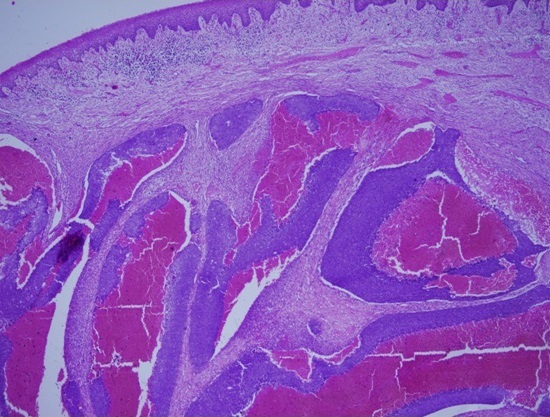
The tumor here displaying mainly a cystic component with central hemorrhage and necrosis

**Figure 4:**
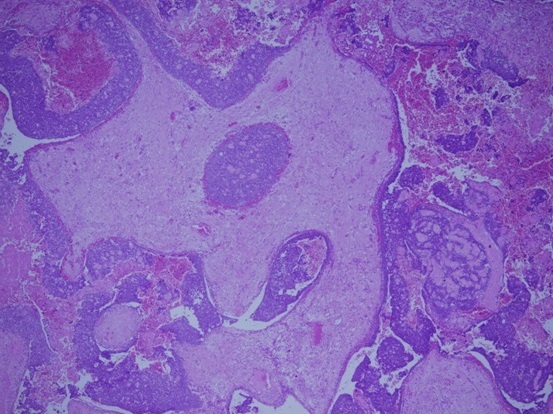
The same tumor displaying both the cystic and solid component

**Figure 5:**
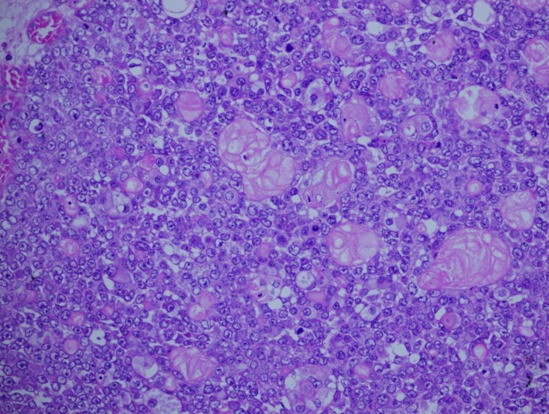
H & E stain the tumor shows the basaloid epithelial element and numerous collections of ghost cells with areas displaying increased mitosis

**Figure 6:**
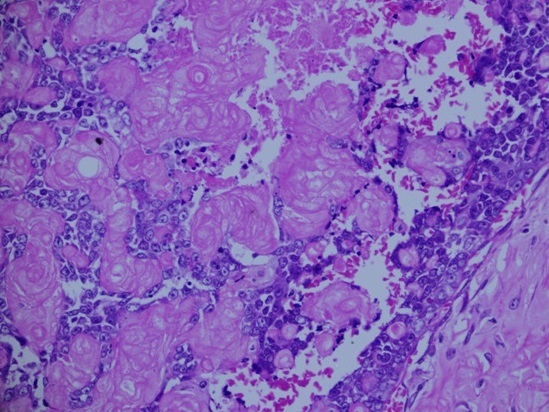
High power image showing an area consisting mainly of ghost cells with few intervening epithelial component

**Figure 7:**
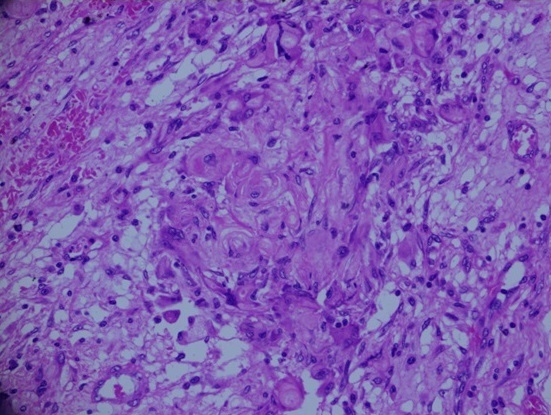
This figure shows numerous ghost cells with foreign body giant cell reaction

Intraorally, a solitary swelling, causing obliteration of left buccal vestibule with overlying indurated mucosa and palatal expansion was noted. No pus or bloody discharge was seen (Figure 1). Computer Tomographic (CT) Scan showed ill-defined mass with scattered radiopacities (Figure 2). No further lesions were detected in CT views, and there was no evidence of metastatic disease on chest radiograph or abdominal ultrasound. Histological examination of the incisional biopsy was suggestive of odontogenic carcinoma. The lesion was excised via an extra-oral approach (modified Weber Fergusson incision) with safety margins. The excisional biopsy showed an epithelial tumor with both cystic and solid components consisting of basaloid ameloblast-like cells displaying peripheral palissading. Numerous collections of ghost cells and areas rich in foreign body type giant cells were noted. The epithelial cells have increased mitotic activity with many abnormal mitotic figures. The surrounding tissue showed fibrosis and bone destruction. The periphery of tumor nests showed neovascularization. The diagnosis of GCOC was made (Figure 3, Figure 4, Figure 5, Figure 6, Figure 7).

One of the surgical margins was involved, namely infrorbial rim, so the patient was subsequently referred to the radio-isotope center for adjuvant radiotherapy and chemotherapy for local control. The patient received external-beam radiotherapy in the form of 3 fields: 2 lateral wedged and one anterior - similar to conventional radiotherapy to the maxilla. The patient received 50 Gray / 25 F / 5 weeks by Linear Accelerator - 6 MV.

Five months later, the patient presented with swelling in the left infra-orbital area. Histological examination of an excisional biopsy revealed an epithelial tumor of the same features of the previous tumor with less ghost cell component. One year later, at the time of reporting the case, unfortunately, the patient passed away and he was disease free.

## Discussion

**GCOC** is an uncommon neoplasm and was first described in 1985 as well documented case report by Ikumra et al [5]. It is considered as malignant variant of odontogenic ghost cell tumor (OGCT) or calcified odontogenic cyst (COC) [6].

As the term “Ghost cell odontogenic carcinoma” underlines, Odontogenic because of ameloblast like cell, Ghost “due to the presence of shadows of keratinized epithelial cells with wet keratin”. Various names were applied to this tumor including malignant COC, Ghost cell odontogenic carcinoma, aggressive epithelial ghost cell odontogenic tumor, dentinogenic ghost cell ameloblastoma, carcinoma arising in a COC, and malignant calcifying ghost cell odontogenic tumor [6].

It is more common in males with an age peak at the fourth decade of life (2). Most of GCOC occurred within the maxilla. Clinical presentation varies with painful swelling and paresthesia being the most common complaint. Facial swelling is also common finding [7]. The geographical distribution of GCOC is prominent in Asia and was attributed to the fact that COC is more common in Asian and the GCOC may arise from the lining of the cyst or malignant transformation of benign tumor [8]. Radiologically, the lesion most commonly seen as a mixed radiolucency and radiopacity with ill defined margins and tooth displacement or root resorption although impacted tooth was reported [9]. Histologically, the tumors that are classified as Ghost cell odontogenic carcinoma may contain both solid and cystic areas although some are completely solid. Microscopically the diagnosis depends on identification of malignant epithelial tumor with classic features of benign calcifying cystic odontogenic tumor. The malignant component of this tumor consists of rounded epithelial islands in a fibrous stroma. The epithelial cells are generally small, rounded with hyperchromatic nuclei or large with vesicular nuclei. Many mitoses are seen. Ghost cells are found in varying numbers either isolated or in clusters [10]. The solid variant shows greater tendency to develop foreign body granuloma.

The treatment of choice is surgical excision with safety margins [8]. The issue of using chemo or radiotherapy is not clear due to rarity of this tumor. However, in the present case, the radiotherapy was not effective. The 5 year survival rate was estimated to be 73%, and the recurrence is common [9]. Therefore, periodic follow up is crucial.

## Conclusion

Ghost cell odontogenic carcinoma is a very rare variant of odontogenic carcinoma. It has an aggressive course of growth in bone. Unlike oral squamous cell carcinoma, the radiotherapy has no role in the treatment of this case and the only effective treatment modality was surgery. This reflects the importance of obtaining clear surgical margins in surgical excision.
